# Cerebral venous thrombosis in a child with nephrotic syndrome: case report

**DOI:** 10.11604/pamj.2012.13.57.1954

**Published:** 2012-11-19

**Authors:** Shalinee Bhoobun, Alhaji Alusaine Jalloh, Kathryn H Jacobsen

**Affiliations:** 1 Department of Medicine, Sheffield Teaching Hospitals, Sheffield, UK; 2 Mercy Hospital, Bo, Sierra Leone; 3 Department of Global and Community Health, George Mason University, Fairfax, Virginia, USA

**Keywords:** nephrotic syndrome, children, cerebral venous thrombosis, Sierra Leone

## Abstract

Cerebral venous thrombosis (CVT), a rare and life threatening complication of nephrotic syndrome, has a variable and non-specific presentation, posing diagnostic challenges. We describe a case of CVT in a Sierra Leonean child with nephrotic syndrome who was successfully treated for the condition despite the resource limitations of the hospital. This case highlights the importance of considering cerebral venous thrombosis as a complication of idiopathic nephrotic syndrome in children presenting with neurological symptoms.

## Introduction

Nephrotic syndrome, which affects about 16 per 100,000 children annually [1], is characterised by a triad of proteinurea, oedema, and hypoalbumenaemia. Damage to the endothelial surface, the glomerular basement membrane, or the podocyte of the glomerular capillary wall alters permeability, allowing for the filtration of proteins into urine. In turn, hypoalbumenaemia lowers the intravascular oncotic pressure, causing translocation of plasma water into the interstitial space and the development of oedema [1]. Idiopathic nephrotic syndrome is subdivided histologically into three types-minimal change (MCNS), focal segmental glomerulorsclerosis, and membranoproliferative glomerulonephritis - with 70-80% of all cases being due to MCNS. Hepatitis B, hepatitis C, HIV, and malaria are well-recognised precipitants of secondary nephrotic syndrome, particularly in the African setting [1]. Cerebral venous thrombosis is a rare complication of nephrotic syndrome and carries a considerable risk of cognitive impairment, sensorimotor and visual deficits, and epilepsy. The exact incidence is unknown, and recent studies suggest this could be because this phenomenon has been under-diagnosed and under-reported in the past [2].

## Patient and observation

A 14-year old boy with no significant past medical history presented to a small district hospital in southern Sierra Leone with a 4 day history of facial puffiness, peripheral pitting oedema, abdominal pains, and reduced urine output. On examination he was afebrile, BP150/110, heart rate 70. He had significant periorbital and facial oedema, pitting oedema from the feet to the knees, a distended abdomen, ascites, and tender hepatomegaly. Lab results showed haemoglobin 10.9 g/dl, packed cell volume 35%, and positive malaria parasites. Urea (14 mg/dl), creatinine (1.0 mg/dl), sodium (137 mmol/L), and potassium (3.7 mmol/L) were normal. Urinalysis, using a urine dipstick, revealed three pluses of proteinurea, which equates to ≥3g urinary protein per day. Lab facilities for the measurement of serum albumin were not available. The diagnosis of nephrotic syndrome was made and the patient was started on a course of prednisolone 60mg/day, Enalapril 2.5mg daily, anti-malarial treatment, and empirical broad spectrum IV antibiotics to cover bacterial infections, in addition to a salt-restricted diet. On day 3 of admission he showed a clinical response to treatment, as the facial oedema, ascites, and BP had reduced. Urinalysis showed two pluses of proteinurea (0.5-1.0 gram per day per day). The child complained of a moderate headache, but had no fever, meningism, or focal neurological signs. He was started on oral paracetamol.

**Figure 1:**
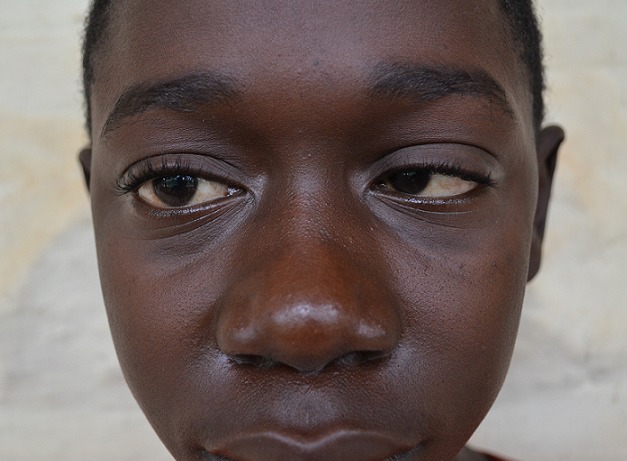
Picture of the child with nephrotic syndrome, demonstrating a typical nephrotic facial puffiness and a right VI nerve palsy. Patient is unable to abduct the right eye. (Written consent to use photograph and case details has been given by the parent and child)

The next morning (day 4 of admission) his symptoms evolved: his headache became severe, he had pain behind both eyes, and he was vomiting. Examination showed a right VI nerve palsy, diplopia, and tinnitus in the right ear, with some hearing loss (Figure 1). He later became drowsy and had a generalised seizure. Diagnosis and commencement of treatment was delayed due to the absence of CT/MRI brain imaging facilities in the local district. In the context of the child's nephrotic status and focal neurological signs, we had a high suspicion of cerebral venous thrombosis, and made a presumptive diagnosis. The child was started on high dose subcutaneous unfractionated heparin daily. This decision was made cautiously, but it was felt that the advantages surpassed the risks. He also received dexamethasone, high flow oxygen, and elevation of the head to 45 degrees, in order to treat features of raised intracranial pressure.

On day 6 of admission his headache had improved and his focal neurological signs began to resolve. He still had a partial right VI nerve palsy and a convergent squint. His oedema resolved completely by day 10 of treatment, and he was discharged on day 18. He continued prednisolone 60mg daily for a total of 6 weeks, followed by a reduced dose of 40mg on alternate days for 6 weeks. At 12 weeks follow-up, his urinalysis showed a trace proteinurea but he remained in remission.

## Discussion

In this setting, a simple urine dip stick showing proteinurea of 3 or 4 pluses confirms the nephrotic range of >3 g urinary protein per day. Traditional regimens of 60 mg/m^2^ daily for 4 weeks followed by 40 mg/m^2^ on alternate days for 4 weeks have been used in the past [3]. A longer (12 week) course using 60 mg/m^2^ prednisone daily for 6 weeks followed by 40 mg/m^2^ on alternate days for 6 weeks, is now in favour [3]. Supporting studies have shown a lower relapse rate at 1 year (36 vs. 62%) for the 12 week versus standard 8 week therapy [4].

About 2 to 5% of children with nephrotic syndrome develop thromboembolic complications such as CVT [5]. Several mechanisms are responsible for the development of a hypercoagulable state, which increases the risk of clot formation. Urinary loss of antithrombin III, protein C, and protein S, which normally have an anticoagulatory action, is coupled with increased production of fibrinogen and coagulation factors, thrombocytosis, and platelet hyperaggregability. Dehydration, diuretic use, and inherited thrombophilias also contribute to an increased risk of thrombosis [6]. There are no definitive laboratory markers predicting the risk of thrombosis, but raised fibrinogen, low antithrombin III, and low protein S point toward a prothrombotic state. Severe hypoalbuminaemia reflects intravascular hypovolaemia and increased blood viscosity, and appears to be the most significant biochemical risk factor for thrombosis [7].

Clinical presentation of cerebral venous thrombosis can be variable, subtle, and non-specific, causing delays in diagnosis. However, signs of raised intracranial pressure, such as headache, vomiting, irritability, lethargy, and papilloedema, are common [5]. Focal neurological deficits such as cranial nerve palsies and hemiparesis are seen less frequently, but are suggestive of CVT. The presentation of our paediatric patient was similar to those described in previous paediatric case reports of focal neurological signs and raised intracranial pressure [2, 6]. The risk of thrombosis is greater at the onset of nephrotic syndrome and during relapses because of the high loss of coagulation factors and acute intravascular volume depletion during these stages of the disease. MRI/magnetic resonance venography has a high sensitivity for diagnosis, and is the imaging mode of choice in children due to its low radiation risk [6]. However, imaging technology is not available in all practise settings, and some diagnoses must therefore be made presumptively.

The benefits and safety of anticoagulation with unfractionated heparin, low molecular weight heparin, and warfarin in cerebral venous thrombosis have been demonstrated even in children with intracranial bleeds [7]. Early commencement of heparin treatment can positively influence clinical outcome by preventing further evolution of the existing thrombus. Unfractionated heparin or low molecular weight heparin for 5 to 7 days followed by warfarin for 3 to 6 months is the current recommendation [8]. Heparin therapy benefits from supplementation with antithrombin III or fresh frozen plasma, where available, to increase its efficiency [8].

## Conclusion

Children with nephrotic syndrome are at increased risk of thromboembolic complications, particularly at the onset of disease or during a relapse, and the presence of neurological symptoms suggests the possibility of cerebral venous thrombosis. Early recognition and diagnosis allow the clinician to commence appropriate anticoagulation therapy and to thereby reduce the morbidity associated with CVT. This case demonstrates that resource-poor health centres have the ability to provide high standards of evidence-based clinical care, with positive outcomes, in complex cases such as a CVT, even when radiological confirmation is not possible. We were able to successfully treat the patient with anticoagulation therapy, reverse his acute clinical presentation, and restore his normal neurological function following presumptive diagnosis based solely on the clinical presentation.
